# Photoreceptor Integrity in MEWDS: Longitudinal Structure-Function Correlations

**DOI:** 10.1167/iovs.65.4.28

**Published:** 2024-04-17

**Authors:** Maria Vittoria Cicinelli, Giovanni Montesano, Alessandro Berni, Pierluigi Scandale, Giovanni Ometto, Ugo Introini, Maurizio Battaglia Parodi, Francesco Bandello, Elisabetta Miserocchi, Alessandro Marchese

**Affiliations:** 1 School of Medicine, Vita-Salute San Raffaele University, Milan, Italy; 2 Department of Ophthalmology, IRCCS San Raffaele Scientific Institute, Milan, Italy; 3 City, University of London, Optometry and Visual Sciences, London, United Kingdom; 4 NIHR Biomedical Research Centre, Moorfields Eye Hospital NHS Foundation Trust and UCL Institute of Ophthalmology, London, United Kingdom; 5 London Northwest University Healthcare NHS Trust, London, United Kingdom

**Keywords:** multiple evanescent white dot syndrome (MEWDS), microperimetry, spectral-domain optical coherence tomography (SD-OCT), photoreceptor reflectivity ratio (PRR), retinal threshold sensitivity (RTS), foveal granularity

## Abstract

**Purpose:**

The purpose of this study was to investigate structure-function correlations in multiple evanescent white dot syndrome (MEWDS) using microperimetry (MP) and spectral-domain optical coherence tomography (SD-OCT).

**Methods:**

Single-center prospective observational study including 14 eyes from 13 patients with MEWDS monitored over a median of 49.5 days (interquartile range = 29–92 days). Investigations focused on best-corrected visual acuity (BCVA), foveal granularity, and the Photoreceptor Reflectivity Ratio (PRR) as a measure of photoreceptor integrity. MP assessed average retinal threshold sensitivity (RTS) and bivariate contour ellipse area (BCEA) for fixation stability. A linear mixed model was used to test associations and interactions among RTS, time, and clinical variables. A hierarchical linear mixed model was used to analyze structure-function relationships, addressing both individual and location-specific variations.

**Results:**

Overall, 2340 MP locations were tested. PRR revealed a transient decrease within 30 days post-presentation, indicative of early photoreceptor disruption, followed by a progressive increase, signaling recovery. Significantly lower foveal sensitivity (RTS = 14.8 ± 7.4 vs. 22.5 ± 4.4 decibel [dB], *P* = 0.04) and increased fixation spread (63% BCEA = 1.26 ± 0.97 vs. 0.48 ± 0.35 deg^2^, *P* = 0.06) were noted in eyes with foveal granularity compared to those without. A significant increase in RTS was demonstrated over time (0.066 dB/day, *P* < 0.001), with a central-to-peripheral gradient of improvement. The interaction between follow-up time and baseline BCVA (*P* < 0.001) indicated more rapid improvement in eyes with worse initial vision. There was a robust, nonlinear association between PRR and RTS across all tested locations (*P* < 0.001), becoming asymptotic for sensitivity losses exceeding 20 dB.

**Conclusions:**

Photoreceptor reflectivity accurately aligned with visual function in MEWDS on longitudinal examinations. The central-to-peripheral gradient of improvement may suggest specific vulnerabilities underlying the area around the disc.

Multiple evanescent white dot syndrome (MEWDS) is a unilateral, occasionally bilateral, inflammatory ocular disorder, primarily affecting young and middle-aged women.^1^ Characterized by distinctive, yellowish, deep retinal lesions, MEWDS causes alterations in the ellipsoid zone (EZ) and the interdigitation zone (IZ) as visualized through spectral-domain optical coherence tomography (SD-OCT).^2^ In some instances, a fundus examination may reveal additional markers like foveal granularity or Jampol dot accompanied by hyper-reflective material beneath the fovea.^3^

Patients with MEWDS typically present with acute visual loss, photopsia, and an enlarged blind spot.^4^ Although visual disturbances often resolve spontaneously with macular integrity restoration, the recovery of visual function may be incomplete^5^ and might not directly correlate with the photoreceptor layers' reappearance on SD-OCT.^6^ Consequently, EZ/IZ disruption indicates disease activity but is not a definitive biomarker for macular function recovery.

Microperimetry (MP) has recently gained importance in establishing precise morpho-functional correlations within the macula, assessing both the location and stability of fixation.^7^^,^^8^ Whereas existing studies have documented relative and absolute scotomata in MEWDS,^9^^–^^11^ a comprehensive topographic analysis of structure-function correlation remains largely unexplored.

This study addresses this gap by longitudinally correlating retinal function, as assessed by MP, with SD-OCT findings in MEWDS, from the initial presentation through the recovery phase. Moreover, it explores the functional implications of various clinical and demographic characteristics to deepen the understanding of MEWDS's clinical spectrum and underlying pathophysiology.

## Methods

This prospective, observational study was conducted at the Department of Ophthalmology, San Raffaele Scientific Institute in Milan, Italy, from 2021 to 2023. Ethical approval was obtained from the local institutional review board (Study ID: OCTA_MIMS v.2, date: August 6, 2021), with all procedures adhering to the tenets of the Declaration of Helsinki. Written informed consent granting permission to analyze clinical data and retinal imaging was secured from all participants before their inclusion in the study.

Individuals were eligible if they exhibited clinical and imaging characteristics indicative of MEWDS, either primary or secondary. Secondary MEWDS was determined when associated with unrelated ocular conditions.^12^^,^^13^ Diagnosis of MEWDS was based on characteristic clinical findings: multifocal white spots on fundus examination, distinctive “wreath-like” hyperfluorescence on fluorescein angiography (FA), hypofluorescent spots on late indocyanine green angiography (ICGA), EZ/IZ disruption on SD-OCT, and increased autofluorescence on fundus autofluorescence (FAF).^14^^,^^15^ Participants were included if they presented within 30 days of symptom onset. We retained the inclusion criterion of up to 30 days from symptom onset to ensure a comprehensive capture of the disease's variable presentation and to accommodate potential delays in patient presentation. Exclusion criteria encompassed any systemic inflammatory, infectious, or neoplastic conditions that could mimic MEWDS, which were ruled out by means of specific diagnostic, laboratory, and imaging tests, and based on clinical presentation and evolution.^16^

Participants underwent a comprehensive ophthalmological assessment, including the measurement of best-corrected visual acuity (BCVA) using decimal charts, slit-lamp biomicroscopy, indirect fundus ophthalmoscopy, and color fundus photography (Optos plc, Dunfermline, Scotland). Multimodal imaging comprised SD-OCT, FAF, FA, and ICGA (Spectralis HRA; Heidelberg Engineering, Heidelberg, Germany). On the same day, macular integrity assessment was conducted using the scanning laser ophthalmoscope (SLO) Microperimeter (MAIA; CenterVue-iCare, Padova, Italy).

After the first visit (baseline), follow-up visits were arranged at 2 weeks, 6 weeks, and 3 months, with a ±1-week window. During these visits, SD-OCT and MP were performed in a follow-up mode to maintain spatial consistency across examinations. In bilateral cases, both eyes were assessed, whereas in unilateral cases, only the affected eye was considered.

### SD-OCT Analysis

A standardized cube scan protocol for SD-OCT imaging consisted of 19 B-scans, each spaced 258 microns apart. Foveal granularity was identified as a localized increase in the retinal pigment epithelium (RPE) layer thickness, along with punctate subretinal accumulations of hyper-reflective material.^3^^,^^17^ The presence of vertical hyper-reflective lines within the fovea^18^ was assessed. The horizontal extent of foveal granularity, when present, and subfoveal choroidal thickness in all eyes were manually measured.

**Figure 1.**
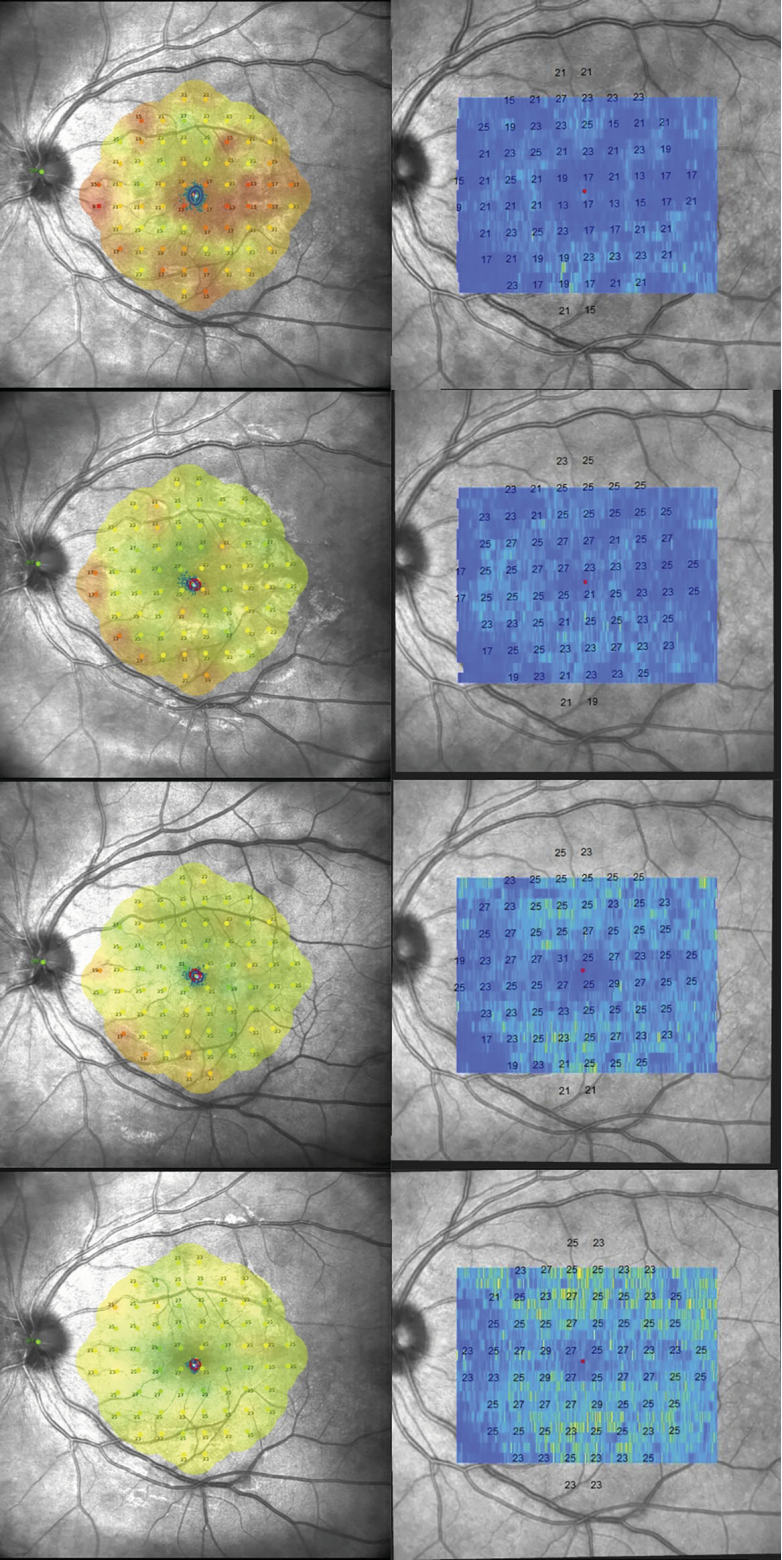
**Sequential functional and structural analysis in a** **patient with** **MEWDS.** This figure visually represents the changes in retinal sensitivity and photoreceptor integrity in a patient with MEWDS across multiple time points: baseline, 2 weeks, 6 weeks, and 3 months. The *left column* is the microperimetry which demonstrates the 68-stimuli pattern covering the central 10 degrees of the retina, assessing the retinal threshold sensitivity (RTS) at various points within the macular region. The resulting sensitivity map provides a detailed overview of the functional status of the macula. The *right column* is the photoreceptor reflectivity ratio (PRR) maps derived from optical coherence tomography. Co-registered over the scanning laser ophthalmoscopy (SLO) fundus image from the microperimetry, these maps offer a visual and quantitative assessment of photoreceptor layer integrity across the retinal volume. Structure-function correlation is obtained for 64/68 central stimuli. The *color-coded* representation shows darker blue areas indicating lower PRR values, suggestive of reduced photoreceptor reflectivity, whereas the *brighter yellow areas* correspond to higher PRR values, indicative of healthier or recovering photoreceptor areas. The RTS values from the microperimetry are superimposed on the PRR map at all examined locations, directly comparing functional sensitivity and structural integrity. Throughout follow-up, an overall increase in PRR values correlating with improvements in retinal sensitivity are noticed.

In our study, the photoreceptor reflectivity ratio (PRR) was calculated as a quantitative index of photoreceptor structural damage. The calculation process involved the measurement of back-reflected light intensity within a specified tissue band, positioned between 20 and 6 pixels above the RPE.^19^ This placement was chosen to approximate the anatomic region of the photoreceptor outer segments, crucial for assessing the integrity of these layers.^20^ This calculated ratio inherently accounted for any variances caused by media opacities, normalizing the reflectivity to adjust for potential optical interferences. A lower PRR value indicated diminished reflectivity, suggesting potential damage. PRR values for each A-scan were then used to create comprehensive PRR maps, providing a visual and quantitative representation of photoreceptor integrity across the scanned retinal volume (Fig. 1).

### Microperimetry

Performed under mesopic conditions after pupil dilation, MP utilized a rectilinear grid of 68 stimuli covering the central 10 degrees, with Goldmann III stimuli, and a 4-2 staircase strategy. The fixation target was set as a 1 degree diameter red circle. Our analysis concentrated on extracting average and foveal retinal threshold sensitivity (RTS; in decibels [dB]). Foveal RTS was determined by averaging the four closest sensitivity values around the central fovea. Fixation stability (FS) was categorized based on the distribution of fixation points relative to the barycenter of the fixation cloud.^21^ The bivariate contour ellipse area (BCEA) was calculated for 63% and 95% of fixation points to quantify the fixation spread. Given the differences in resolution and focal areas between the SD-OCT scans and the macular integrity assessment (MAIA) grid, an affine transformation technique was used to align functional data from MP with structural imaging from SD-OCT using the R NiftyReg package^22^; structure-function correlation was obtained for 64 of 68 central stimuli.

### Statistical Analysis

Continuous variables were summarized as mean ± standard deviation (SD) or median and interquartile range (IQR). Categorical variables were reported as relative percentages. Spaghetti plots were utilized to visualize the trends of quantitative variables over time. Additionally, as part of our initial exploratory analysis, correlation plots were generated to display Pearson correlation coefficients (r) and relative *P* values.

Inferential statistics were primarily directed at understanding the associations of MP parameters and their longitudinal changes. Mixed-effects models were utilized to account for repeated measures and the correlation of data within the same eye or the same patient. Potential interactions among RTS, time, and clinical variables, such as age, gender, or presenting BCVA, were also assessed.

A scaling framework by Hood and Kardon was adopted to examine the relationship between structural changes observed on SD-OCT and functional outcomes from MP.^23^ This involved converting dB sensitivity metrics into a linear scale (linear sensitivity = 10^ [dB/10]) to facilitate direct comparison between structural and functional measurements. These relationships were modeled with linear regressions, constructing a two-level hierarchical structure to address location-specific and subject-specific variations in structure-function relationships. All statistical analyses were performed using the statistical software R (R Foundation for Statistical Computing, Vienna, Austria).

## Results

Our study included 14 eyes from 13 patients, predominantly female patients (79%) with an average age of 32.5 ± 12.7 years. Primary MEWDS was diagnosed in 79% of the cases, with these patients being notably younger (28 ± 7.1 years) and less myopic (−0.72 ± 1.97 diopters) than patients with secondary MEWDS (49 ± 16.52 years and −3.95 ± 2.05 diopters, respectively; Table 1). The median duration from symptom onset to presentation was 6.5 days (IQR = 2.25–13 days). BCVA at presentation ranged from 0 to 1 LogMAR, with a moderate inverse correlation observed with refraction (*r* = −0.53, *P* = 0.06; Supplementary Fig. S2).

**Table 1.** Demographic and Clinical Characteristics of Patients With MEWDS

	Overall (*N* = 14 Eyes)
Age, y	
Mean (SD)	32.5 (12.7)
Median [min, max]	28.5 [20, 60]
Female gender	10 (77%)
Time from symptoms onset, d	
Mean (SD)	9.79 (10.1)
Median [min, max]	6.50 [1, 30]
Primary MEWDS	11 (79%)
Refraction, diopters	
Mean (SD)	−1.22 (2.25)
Median [min, max]	−0.25 [−5.40, 1.00]
BCVA, LogMAR	
Mean (SD)	0.25 (0.27)
Median [min, max]	0.20 [0, 1.00]
Foveal granularity	7 (50%)
Fovea granularity size, µm	
Mean (SD)	751 (458)
Median [min, max]	550 [265, 1500]
Choroidal thickness, µm	
Mean (SD)	350 (93.3)
Median [min, max]	325 [219, 528]
Vertical hyper-reflective line	4 (29%)
Photoreceptor reflectivity ratio	
Mean (SD)	6.58 (1.26)
Median [min, max]	6.71 [4.66, 8.58]

BCVA, best-corrected visual acuity; PRR, photoreceptor reflectivity ratio.

Data are presented as mean (standard deviation) and median [minimum − maximum] for continuous variables, and counts (percentages) for categorical variables.

No treatment was administered to patients with primary MEWDS. Among those with secondary MEWDS, one individual with angioid streaks received no treatment, whereas two patients with punctate inner choroidopathy were prescribed oral corticosteroids. Out of the total, two eyes were only available for baseline examination and did not contribute to longitudinal data. Two eyes were excluded due to bilateral progression: in the right eye, the spots spread from the mid- to the far-periphery, and in the left eye, initially localized spots in the peripapillary region extended to cover the entire posterior pole (Supplementary Fig. S1). The remaining cohort was followed for a median duration of 49.5 days (IQR = 29–92 days), with all eyes achieving 20/20 vision by the final follow-up visit.

### SD-OCT Changes

Half of the eyes exhibited foveal granularity, with their horizontal size averaging 751 ± 458 µm. A negative correlation emerged between the size of foveal granularity and a shorter duration of the disease (*r* = −0.72, *P* = 0.07; see Supplementary Fig. S2). A significant decrease in the PRR was observed initially (12 to 30 days post-presentation) in 7 out of 10 eyes (70%), followed by a gradual increase (Supplementary Fig. S3).

### Microperimetry Testing

Overall, 2340 MP locations were tested. At baseline, the average RTS was 18.1 ± 6.2 dB, with foveal sensitivity slightly higher at 18.7 ± 7.1 dB. Stable fixation was observed in all but one eye (7%), which had the lowest RTS of 4.9 dB and the highest 63% and 95% BCEA values (3.1 and 9.2 deg², respectively). Notably, a pronounced inverse relationship was observed between fovea sensitivity and BCEA (*r* = −0.70, *P* < 0.01), suggesting that increased central sensitivity correlated with more focused fixation.

Significant linear relationships were established between baseline RTS and the time elapsed from symptom onset (*r* = 0.56, *P* = 0.02) as well as the presenting BCVA (*r* = −0.73, *P* = 0.002). All scatter plots illustrating correlations between continuous variables are presented in Supplementary Figure S2.

Foveal granularity was associated with significantly lower foveal sensitivity (*P* = 0.04) and a broader fixation spread (*P* = 0.06), indicating its impact on visual function despite not affecting the overall macular RTS (Table 2).

**Table 2.** Comparison of Microperimetry Baseline Parameters in Patients With MEWDS With and Without Foveal Granularity

	Foveal Granularity			
	No (N = 7)	Yes (N = 7)	Overall (N = 14)	*P* Value
Average RTS, dB				
Mean (SD)	18.5 (6.0)	17.6 (6.8)	18.1 (6.2)	0.7
Median [min, max]	18.4 [10.0, 25.6]	20.1 [4.90, 24.9]	19.8 [4.90, 25.6]	
Foveal RTS, dB				
Mean (SD)	22.5 (4.4)	14.8 (7.4)	18.7 (7.1)	0.04
Median [min, max]	23.0 [14.0, 26.5]	16.5 [4.25, 25.5]	19.4 [4.25, 26.5]	
Fixation stability (%)				
Stable	7 (100)	6 (86)	13 (93)	0.9
Relatively unstable	0 (0%)	1 (14%)	1 (7%)	
BCEA 63, deg^2^				
Mean (SD)	0.48 (0.35)	1.26 (0.97)	0.86 (0.82)	0.06
Median [min, max]	0.30 [0.20, 1.20]	0.90 [0.20, 3.10]	0.55 [0.20, 3.10]	
BCEA 95, deg^2^				
Mean (SD)	1.31 (1.04)	3.73 (2.88)	2.52 (2.43)	0.06
Median [min, max]	0.80 [0.60, 3.50]	2.80 [0.70, 9.20]	1.60 [0.00, 9.20]	

BCEA, bivariate contour ellipse area for 63% and 95% of fixation points; RTS, retinal threshold sensitivity.

Data are presented as mean (standard deviation) and median [minimum − maximum] values. The statistical significance between groups, calculated with linear mixed models, is represented by *P* values.

**Figure 2.**
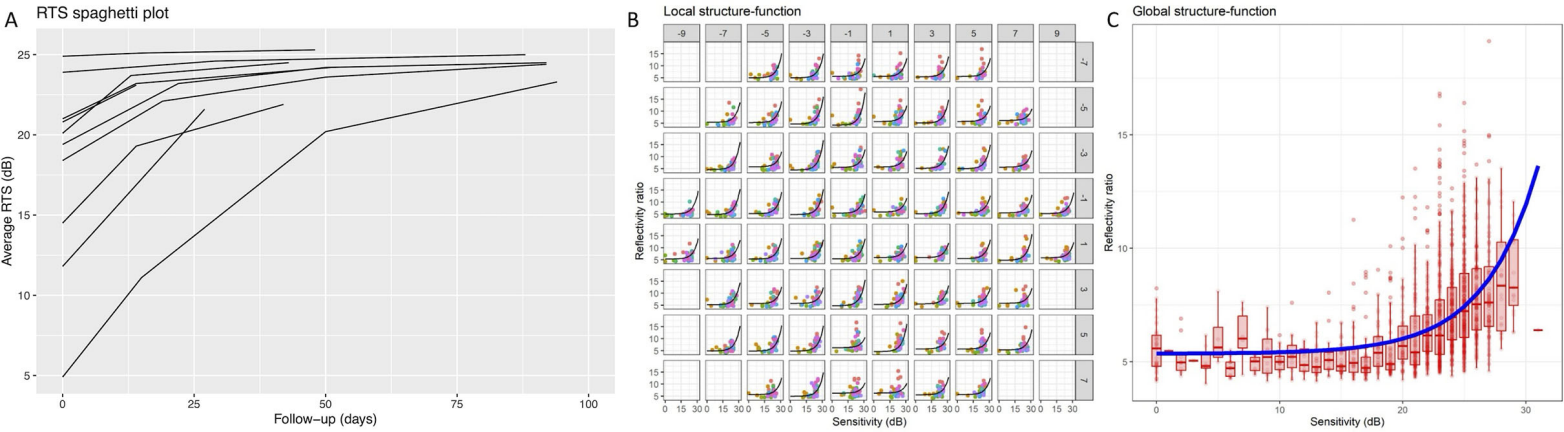
**Retinal threshold sensitivity (RTS) and structure-function relationship in MEWDS.** (**A**) Spaghetti plot of retinal threshold sensitivity (RTS) over time for each eye, measured in decibels (dB), illustrating the trajectory of visual function recovery. (**B**) Local analysis depicting the correlation between photoreceptor reflectivity ratio (PRR) as observed on optical coherence tomography (OCT) and corresponding retinal sensitivity at each specific test location as measured by microperimetry. (**C**) Global analysis showing the aggregate correlation between overall PRR on OCT and the averaged retinal sensitivity from all test locations on microperimetry, reflecting the average structure-function relationship in MEWDS.

A significant increase in RTS of 0.066 dB per day (*P* < 0.001) was observed, illustrating an improvement to 21.7 dB ± 4.07 at 2 weeks, 23.5 dB ± 1.64 at 6 weeks, and 24.4 dB ± 0.67 at 3 months (Fig. 2A). The recovery pattern displayed a centrifugal trend, initiating from the central macula and extending outward to the extramacular regions and mid-periphery, finally reaching the optic disc area. However, whereas all patients demonstrated this general trend of improvement, the extent and visibility of these changes over time varied among individuals. Specifically, patients presenting with less profound scotomas exhibited less pronounced changes in their improvement patterns over the observed period.

**Figure 3.**
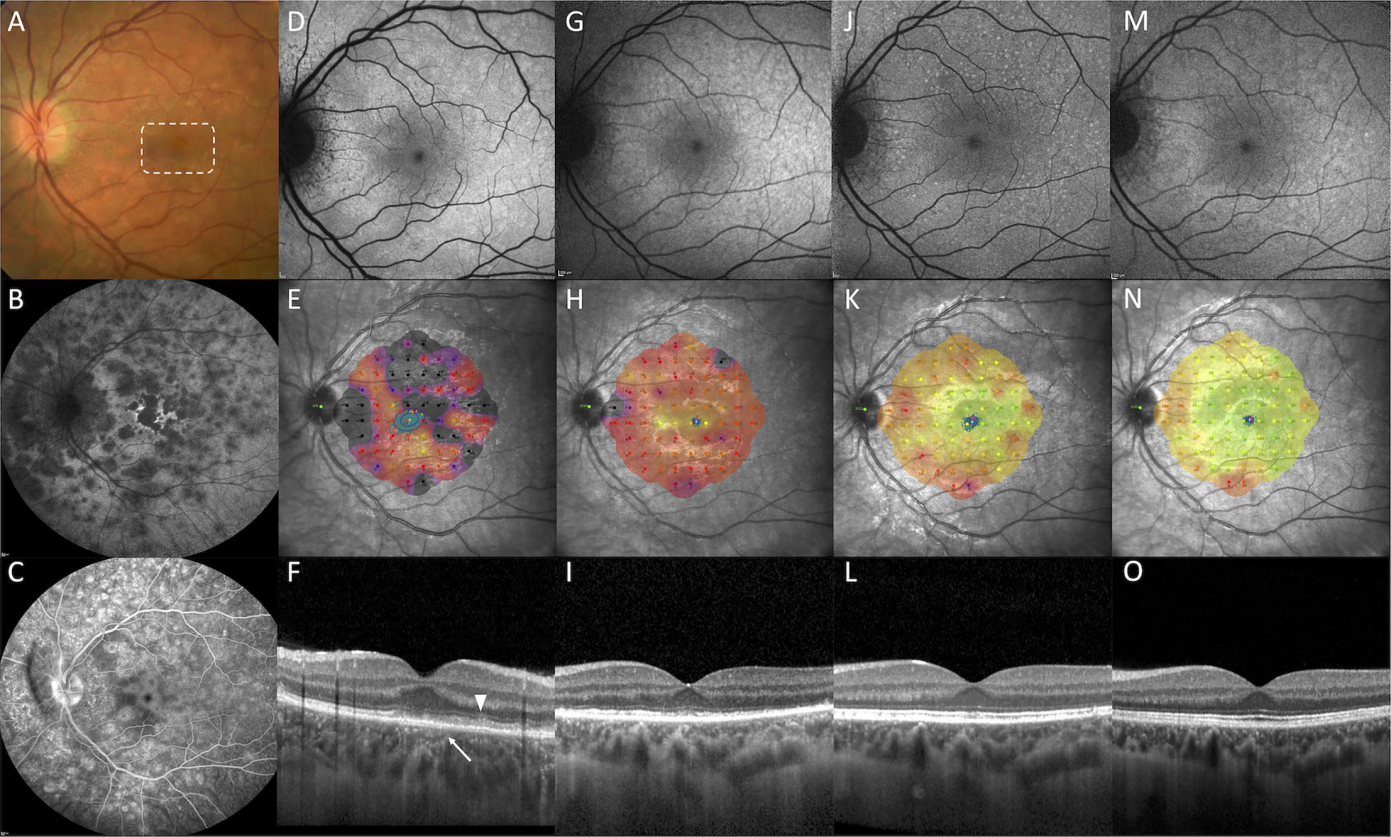
**Comprehensive multimodal imaging and functional assessment of a** **patient with** **MEWDS** **over time.** (**A**) The baseline color fundus photograph exhibits faint *white-yellow spots* characteristic of MEWDS and central foveal granularity (*white rectangle*). (**B**) The baseline late phase indocyanine green angiography (ICGA) reveals the hypofluorescent spots and dots around the posterior pole, mid-periphery, and around the disc, including a prominent spot in the fovea with a hyperfluorescent halo. (**C**) The baseline late-phase fluorescein angiography (FA) displays wreath-like hyperfluorescence, leakage from the optic disc and large venules. (**D**) The baseline blue-light fundus autofluorescence (FAF) shows hyperautofluorescent spots correlating with those seen in color fundus and ICGA. (**E**) The baseline microperimetry indicates a global reduction in retinal threshold sensitivity (RTS) with darker areas representing deeper scotomata. Absolute scotoma areas correspond closely to the hypofluorescent spots on ICGA, and less precisely to the FAF spots. The *blue points* represent the cloud of points describing the preferred retinal locus (PRL), and the *purple lines* illustrate the bivariate contour ellipse area (BCEA), indicative of fixation spread. At baseline, both the PRL and BCEA are spread out, suggesting a less focused fixation area. (**F**) Baseline optical coherence tomography (OCT) shows diffuse disruption of the ellipsoid zone (EZ) and the interdigitation zone (IZ) with mild RPE thickening (*arrow*). Small projections of hyper-reflective material extend toward the inner retina crossing the external limiting membrane (*arrowhead*). (**G**) The 2-week FAF demonstrates a gradual fading of hyperautofluorescent spots. (**H**) The 2-week microperimetry shows improvement in macular sensitivity, more marked in the central fovea, with persistent deeper scotomata in the extramacular and peripapillary regions. The BCEA becomes markedly smaller, and the cloud of PRL more concentrated around the fovea. (**I**) The 2-week OCT reveals thinning of the RPE, persistent diffuse multifocal damage of the EZ, absence of the IZ, and disappearance of the hyper-reflective subretinal material. (**J**) The 6-week FAF shows the disappearance of the original hyperautofluorescent spots replaced by tiny hyperautofluorescent dots. (**K**) The 6-week microperimetry demonstrates a further improvement in macular sensitivity with a noticeable centrifugal pattern of improvement. (**L**) The 6-week OCT exhibits almost complete restoration of the EZ and partial reconstitution of the IZ. (**M**) The 3-month FAF shows almost complete normalization of the autofluorescence signal. (**N**) The 3-month microperimetry indicates normalization of foveal sensitivity with persistent relative scotomata in the extramacular and peripapillary regions. (**O**) The 3-month OCT demonstrates complete reconstitution of both the EZ and IZ.

**Figure 4.**
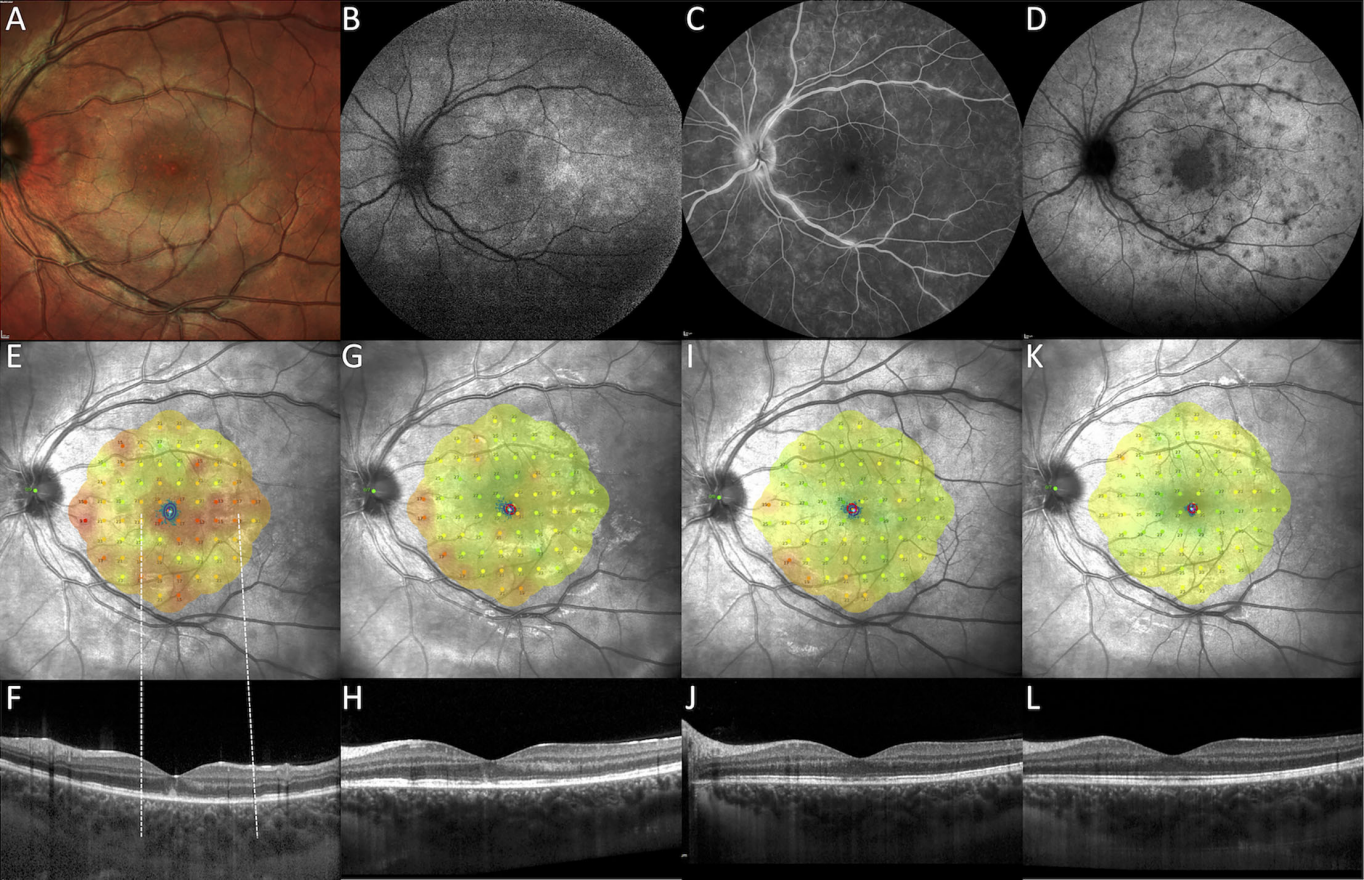
**Longitudinal multimodal imaging and microperimetry in a** **patient with** **MEWDS.** (**A**) The baseline Multicolor fundus photograph reveals irregular retinal reflectivity with tiny hypopigmented spots in the macula, indicative of foveal granularity (*white rectangle*). (**B**) The baseline fundus autofluorescence (FAF) shows hyperautofluorescent spots predominantly in the mid-periphery, suggesting active inflammatory lesions. (**C**) The baseline fluorescein angiography (FA) displays characteristic wreath-like hyperfluorescence and vascular leakage. (**D**) The baseline indocyanine green angiography (ICGA) exhibits hypofluorescent spots with a prominent hypofluorescent plaque in the macula. (**E**) The baseline microperimetry illustrates significantly reduced retinal sensitivity in the macula and peripapillary region, with relative preservation in the perifoveal area. The spread of fixation, as indicated by the 63% and 95% bivariate contour ellipse area, is broad and predominantly vertical. (**F**) The baseline horizontal OCT scan shows diffuse loss of the ellipsoid zone (EZ) in areas correlating with reduced sensitivity (*dashed lines*) and accumulation of hyper-reflective, ill-defined material beneath the fovea. (**G–L**) The follow-up MP and OCT at 2 weeks, 6 weeks, and 3 months demonstrates a gradual improvement in retinal sensitivity, following a characteristic centrifugal pattern, and a corresponding restoration of the EZ/IZ bands.

In fact, RTS improvement was more rapid in eyes with poorer initial BCVA, as indicated by the significant interaction between follow-up time and baseline BCVA (*P* = 0.001) derived from our mixed-effects model analysis (Figs. 3, 4). Other variables, including the classification into primary and secondary MEWDS, were not associated with significant interactions (*P* > 0.05).

### Morpho-Functional Correlations

Our findings revealed a robust association between average RTS and PRR across all examined locations (*P* < 0.001), emphasizing a crucial link between photoreceptor reflectivity seen on SD-OCT and functional outcomes from MP. The global Hood and Kardon model was estimated as follows:

$$\mathrm{PRR}=5.36+(0.66*10^{(-2)})*\mathrm{Sensitivity}\_\mathrm{linear}.$$

In addition, this confirmed the uniformity of this relationship across the posterior pole with no notable variation in slope due to eccentricity (*P* = 0.7; Figs. 2B, 2C). The significance of this model persisted even when accounting for varied nesting of random effects (*P* = 0.001).

### Correlations With Other Imaging Modalities

The study observed frequent overlaps between areas of relative scotoma and hypofluorescent spots on ICGA and EZ/IZ disruption on SD-OCT. On the other hand, the correlation with hyperautofluorescence on FAF imaging was less precise, especially during the recovery phase. Despite improvements or normalization in the posterior pole FAF, indicative of structural recovery of photoreceptors,^24^ various regions persistently exhibited reduced retinal sensitivity (see Figs. 3, 4).

## Discussion

This study provided a detailed examination of the morpho-functional relationships in MEWDS by using MP and SD-OCT. Our results highlighted a clear connection between changes in photoreceptor structure and visual function. They also emphasize the importance of identifying foveal granularity and understanding the recovery patterns of photoreceptors in this disease.

MEWDS is part of the broad and heterogenous group of white dot syndromes.^1^^,^^14^ Despite its typical demographic and clinical presentations, our study supports the existing literature, indicating a wide spectrum of clinical manifestations.^25^ The age and refractive errors varied largely among our cohort, probably accounting for the differences between patients with primary and secondary MEWDS.^25^ Consistent with earlier studies, our cohort also presented diversity in visual acuity at onset.^5^^,^^26^ The integration of MP into our investigation enhanced the comprehension of functional deficits in this disease.

MP disclosed profound scotomata surrounded by a broader reduction in overall sensitivity. Interestingly, fixation stability was maintained even in cases where the retinal sensitivity was markedly diminished. This observation suggests a relative preservation of central vision, aligning with electroretinogram findings that indicated both cone and rod response reductions, but a more pronounced impairment in the latter*.**^27^* Nevertheless, half of our patient cohort had presenting BCVA of 20/32 or better, which may partly account for the relatively good functional performance observed in our study.

We explored clinical and demographic associations with retinal sensitivity. Foveal granularity had detrimental effects on central visual function and a wider fixation spread. Additionally, the size of foveal granularity decreased with increasing time from the onset of symptoms, suggesting it may be a potential indicator of early disease. Conversely, the detection of vertical hyperreflective lines, which might signify Muller cell activation in MEWDS,^18^ did not significantly alter retinal sensitivity measures, indicating that the photoreceptors bear the greatest brunt of the disease's impact. Other features, including the presence of primary or secondary MEWDS, did not significantly affect the presenting retinal function or its recovery pattern, suggesting a stereotyped response regardless patients’ characteristics.

This study corroborates the self-limiting nature of MEWDS. We observed that the average RTS improved both in eyes that received corticosteroid treatment for secondary conditions associated with MEWDS, such as punctate inner choroidopathy, and in those that did not receive any treatment, including all cases of primary MEWDS. The functional recovery was more pronounced in eyes with worse initial BCVA, suggesting a potential ceiling effect for those with better initial vision. One of the most interesting findings was a distinctive centrifugal recovery pattern that was consistent across patients, with a faster restoration in the central macula, progressing to the extramacular and peripheral regions, and finally culminating around the optic disc. The extended recovery time of peripapillary scotomas may suggest particular susceptibility of the peripapillary region to inflammation, a feature shared with other white spot syndromes.^28^^–^^30^ This preference for the peripapillary region also raises questions about its vulnerability, whether due to local vascular differences, local photoreceptor or RPE characteristics, or unique structural variations. The possible mechanisms behind this predisposition remain to be elucidated.

Previous OCT and angiographic studies have established the photoreceptor layer as the primary site of damage in MEWDS,^6^^,^^27^ an understanding that is complemented by Gaudric's examination of the “epitheliopathy” theory.^31^ This theory posits that dysfunction within the RPE may precipitate the initial damage to photoreceptors seen in MEWDS. However, microperimetry, while valuable, does not assess the function and integrity of RPE cells directly. These considerations highlight the critical need for using techniques specifically designed to probe RPE function and its interactions with photoreceptors and the choroidal environment in MEWDS, to validate previous conjectures.

The study's findings add to the literature demonstrating a distinct sequential pattern in photoreceptors’ reflectivity, with a transient decrease and then a gradual recovery. This pattern possibly reflects the accumulation of hyper-reflective subretinal material of varying sizes and shapes in the early phase,^2^ which rapidly disappears in the sub-acute phase. Our study used PRR as a quantitative measure of photoreceptor anatomic integrity. PRR turned out to be also a reliable indicator of photoreceptor function, aligning with studies on macular telangiectasia type 2,^32^ age-related macular degeneration,^19^^,^^33^ retinal dystrophies, and other vitreoretinal diseases.^34^ On the other hand, the presence of the EZ band did not conclusively predict the functional status.^35^ In fact, the EZ band reappearance did not correlate well with functional recovery, and this agrees with observations done using Early Receptor Potential (ERP), where prolonged regeneration kinetics were noted in the recovery phase of MEWDS, despite normalizing the fundus appearance.^27^

Notably, the structure-function relationship between PRR and RTS was nonlinear, becoming asymptotic for sensitivity losses exceeding 20 dB. This floor effect suggests that small changes in the PRR were associated with wide variation in retinal sensitivity,^23^ and further accentuates that structural integrity, although critical, may not fully capture the patient's visual function. This complexity highlights the necessity for a combined approach using both structural and functional assessments in managing patients with MEWDS.

The strengths of our study include its prospective nature and the application of advanced structural-functional analysis techniques. However, the limitations, such as the small sample size and short follow-up period, restrict the extrapolation of our findings to a broader MEWDS population. Additionally, whereas our study is insightful, its observational nature limits the ability to make definitive conclusions about the disease's progression and treatment effects. Challenges in standardizing EZ reflectivity measurements and understanding their correlation with underlying photoreceptor structure also necessitate further research.^36^ Whereas incorporating MP into our study on MEWDS offered valuable insights into structure-function correlations, it also highlighted inherent limitations. Predominantly conducted under mesopic conditions, microperimetry does not allow for the isolation of the photoreceptor system responsible for detection thresholds, blurring the distinction between rod and cone contributions.^37^ Its use of predefined grid patterns may inadequately represent the individual variability and spatial heterogeneity of scotomas, particularly in diseases like MEWDS. Moreover, the accuracy of microperimetry is contingent upon patient cooperation and stable fixation, challenging in cases of significant vision loss, unstable fixation, or among young patients.^38^

In conclusion, our study adds valuable knowledge to understanding MEWDS, characterizing the relationship between photoreceptor integrity and visual function. The findings, particularly regarding the patterns of photoreceptor damage and recovery, as well as the implications of foveal granularity, provide a foundation for future research aimed at elucidating the pathophysiology of MEWDS. Our findings also revealed a distinctive centrifugal pattern of visual improvement. This pattern, alongside the predilection for the peripapillary region, prompts further investigation into the specific vulnerabilities underlying the area around the disc.

## Supplementary Material

Supplement 1

Supplement 2

Supplement 3
