# Supplementary material for: Photoreceptor Integrity in MEWDS: Longitudinal Structure-Function Correlations
Source: Invest Ophthalmol Vis Sci. 2024 Apr 17;65(4):28. doi: 10.1167/iovs.65.4.28 (PMC11033598; doi:10.1167/iovs.65.4.28)
Supplement: Supplement 1 [file iovs-65-4-28_s001.pdf]

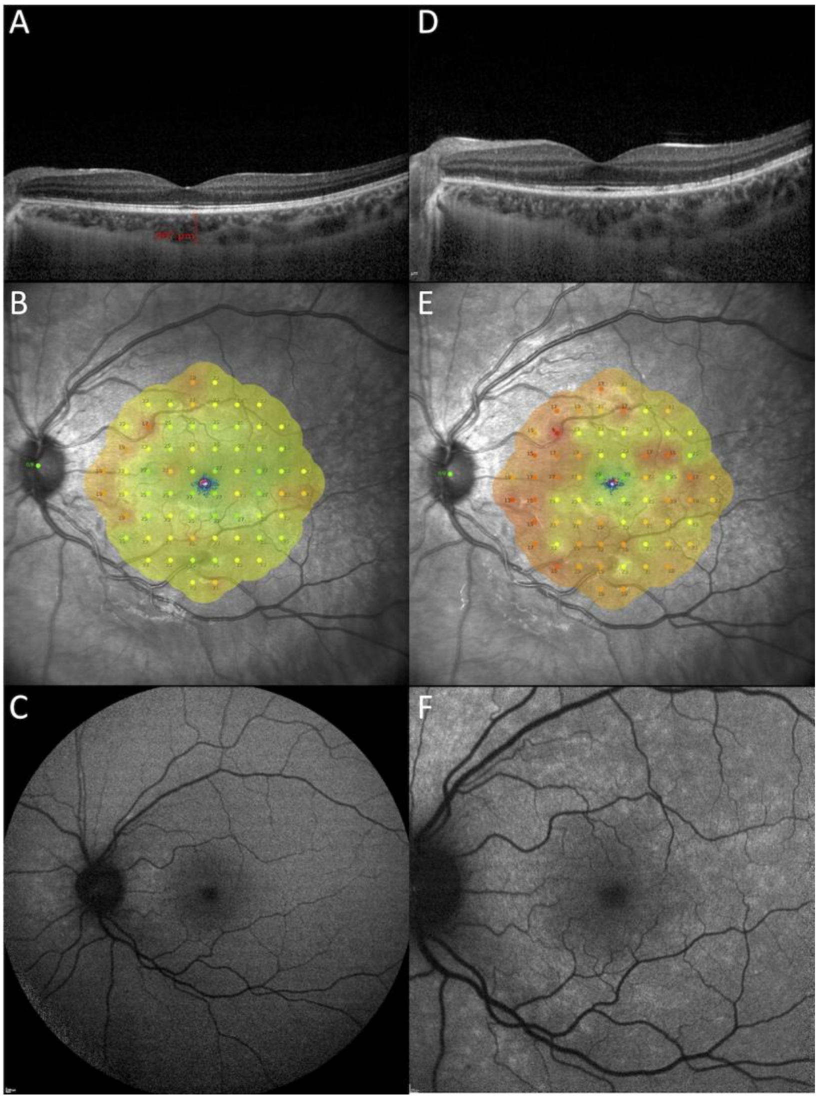

Supplementary Figure 1. Multimodal Imaging and Functional Assessment of a MEWDS Patient with Bilateral Progression.

The patient exhibited an atypical bilateral progression, with the left eye initially showing localized spots in the peripapillary region that extended to cover the entire posterior pole.

A. Baseline Optical Coherence Tomography (OCT) displays disruption of the ellipsoid zone/interdigitation zone (EZ/IZ) bands in the peripapillary area (arrowhead).

B. Baseline microperimetry reveals relative functional scotoma in the peripapillary area and preservation of retinal sensitivity at the posterior pole.

C. Baseline blue-light autofluorescence (FAF) demonstrates hyperautofluorescent spots around the disc, which are more concentrated in the nasal area.

D. Ten days after presentation, the patient reported worsening symptoms and increased photopsia. OCT displayed diffuse disruption of the EZ/IZ bands (awwors).

E. Microperimetry revealed an expansion of reduced sensitivity with a temporal shift toward the macula, approaching the disc.

F. FAF showed diffuse involvement of the posterior pole.
