# Supplementary material for: Photoreceptor Integrity in MEWDS: Longitudinal Structure-Function Correlations
Source: Invest Ophthalmol Vis Sci. 2024 Apr 17;65(4):28. doi: 10.1167/iovs.65.4.28 (PMC11033598; doi:10.1167/iovs.65.4.28)
Supplement: Supplement 2 [file iovs-65-4-28_s002.pdf]

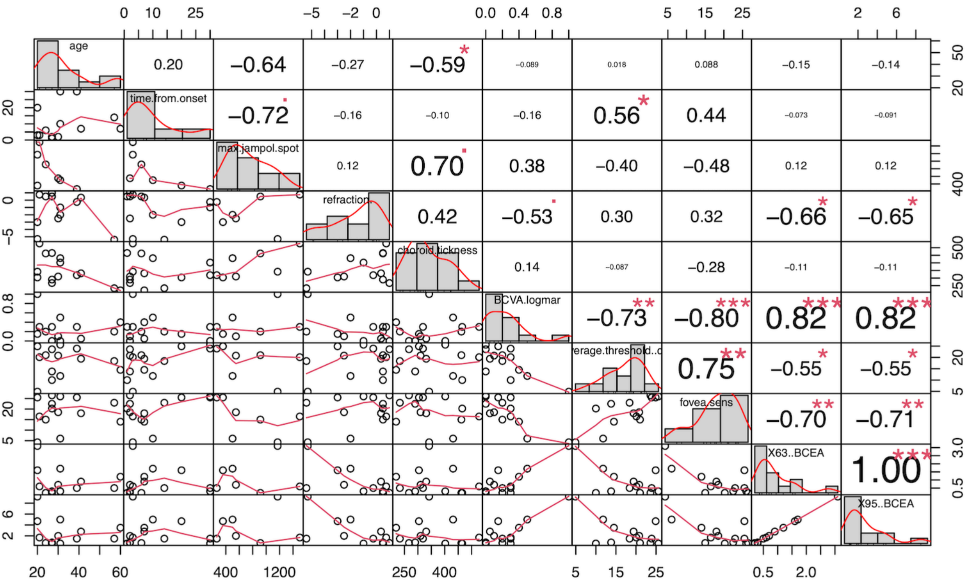
age
time.from.onset
max.jampol.spot
refraction
choroid.tickness
BCVA.logmar
erage.threshold..d
fovea.sens
X63..BCEA
X95..BCEA
0.20
−0.64
−0.27
−0.59 *
−0.089
0.018
0.088
−0.15
−0.14
−0.72 .
−0.16
−0.10
−0.16
0.56 *
0.44
−0.073
−0.091
0.12
0.70 .
0.38
−0.40
−0.48
0.12
0.12
0.42
−0.53 .
0.30
0.32
−0.66 *
−0.65 *
0.14
−0.087
−0.28
−0.11
−0.11
−0.73 **
−0.80 ***
0.82 ***
0.82 ***
0.75 **
−0.55 *
−0.55 *
−0.70 **
−0.71 **
1.00 ***

Supplementary Figure 2: Inter-Variable Correlation Matrix in MEWDS Study

This figure presents a matrix of correlation coefficients among various quantitative measures in the MEWDS study. Each panel showcases scatter plots with fitted regression lines for pairwise comparisons of the study variables. Along the diagonal, histograms visualize the distribution of each individual variable. The axes of the figure correspond directly to the variables included in our correlation matrix, with each axis representing a specific variable. Red stars indicate the significance levels of the correlations (square: <0.1, one asterisk: <0.05; two asterisks: <0.01; three asterisks: <0.001).

Key correlations include:

- Best-Corrected Visual Acuity (BCVA) at presentation in LogMAR, which inversely correlates with the degree of refractive error (r = -0.53, p = 0.06).

- A significant inverse relationship between the size of foveal granularity and the disease duration, indicating larger spots are associated with a shorter disease course (r = -0.72, p = 0.07).

- Foveal sensitivity expressed as Retinal Threshold Sensitivity (RTS, decibel) showing a strong negative correlation with the Bivariate Contour Ellipse Area (BCEA), where lower sensitivity aligns with a broader fixation area (r = -0.70, p < 0.01).

- A notable direct relationship between average baseline RTS and the time from symptom onset as well as initial BCVA, with shorter disease duration and worse initial vision linked to lower RTS (p=0.02 and p=0.002, respectively).
