# Supplementary material for: Photoreceptor Integrity in MEWDS: Longitudinal Structure-Function Correlations
Source: Invest Ophthalmol Vis Sci. 2024 Apr 17;65(4):28. doi: 10.1167/iovs.65.4.28 (PMC11033598; doi:10.1167/iovs.65.4.28)
Supplement: Supplement 3 [file iovs-65-4-28_s003.pdf]

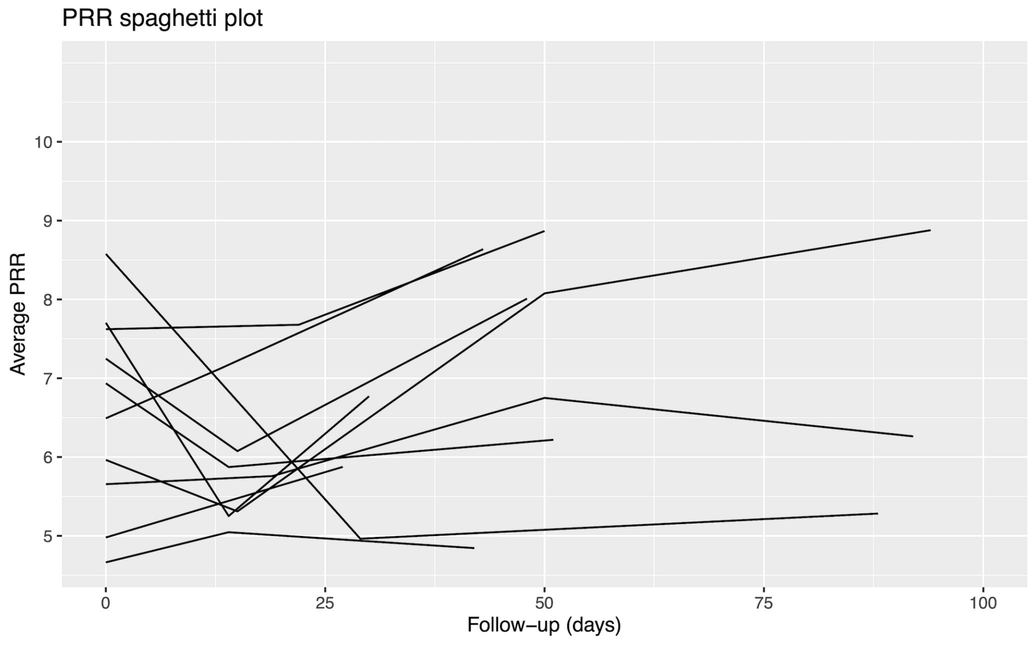

PRR spaghetti plot
Average PRR
10
9
8
7
6
5
0
25
50
75
100
Follow−up (days)

Supplementary Figure 3. Photoreceptor Reflectivity Ratio (PRR) Trajectory Over Time.

This spaghetti plot depicts the average PRR for each patient throughout the study duration. Each line represents the PRR trend of an individual eye, tracking the photoreceptor recovery process. Initial decreases followed by subsequent increases in PRR are visible, corresponding to the dynamic changes in photoreceptor integrity post-MEWDS presentation. The x-axis marks the days of follow-up, while the y-axis indicates the PRR values, with the average PRR shown to improve over time, aligning with the clinical observations of visual function restoration.
